# Knowledge, attitude and practice of endodontic radiographic techniques among dentists: A survey

**DOI:** 10.6026/973206300211093

**Published:** 2025-05-31

**Authors:** Vinod Balla, Saurav Bathla, Ravindra Reddy Regalla, Ankit Anand, Kumari Neha, Pratik Agrawal, Anukriti Kumari

**Affiliations:** 1 Department of Dentistry, Rajiv Gandhi Institute of Medical Sciences, Adilabad, Telangana, India; 2 Department of Conservative Dentistry and Endodontics, Teerthanker Mahaveer Dental College and Research Centre, Teerthanker Mahaveer University, Moradabad, Uttar Pradesh, India; 3 Department of Dental Surgery, Rajiv Gandhi Institute of Medical Sciences (RIMS), Adilabad, Telangana, India; 4 Department of Conservative Dentistry and Endodontics, Mithila Minority Dental College and Hospital, Darbhanga, Bihar, India; 5 Department of Dentistry, Santosh Dental College, Ghaziabad, Uttar Pradesh, India; 6 Department of Conservative Dentistry and Endodontics, Kalinga Institute of Dental Sciences, KIIT Deemed to be University, Bhubaneswar, Odisha, India; 7 Department of Oral and Maxillofacial Radiology, School of Dental Sciences, Sharda University, Greater Noida, Uttar Pradesh, India

**Keywords:** ALARA principle, CBCT, endodontics, radiographic techniques, RVG

## Abstract

The knowledge, attitude, and practice (KAP) of endodontic radiographic techniques among 384 dental practitioners, including general dentists, endodontists, and postgraduate students in India is of interest. While all participants were aware of RVG radiography, only 58.9% were familiar with CBCT applications, reflecting limited exposure and training in advanced imaging. A majority demonstrated positive attitudes toward radiographic use and expressed willingness to pursue further training; though actual CBCT usage was low (39.6%) and protective measures were inconsistently applied. The findings highlight the need for enhanced education and standardized protocols to improve the adoption and safe use of advanced radiographic technologies in endodontic practice. Incorporating emerging technologies like artificial intelligence into training curricula could further enhance diagnostic accuracy and patient safety.

## Background:

Endodontic treatment is a part of the dental practice that comprises specific diagnosis, organization and treatment monitoring [1]. Radiographic analysis remains an essential part of endodontic care, as it is used to visualize root canal anatomy, locate periapical disease, disclose root fractures and evaluate treatment results. Even with technological and digital systems, the effectiveness of endodontic radiographic methods is still very much reliant on how well the clinician knows, thinks about and uses them [2]. Pre-, intra- and Post-Operative Radiographs and root canal treatment radiographs help us make the right decisions and avoid procedural errors. Conventional IOPA radiographs still form the backbone of endodontic imaging and new methods like digital radiography and CBCT have advanced its diagnostic potential. Nevertheless, these modalities' choice and appropriate use depend on the dentist's training, knowledge of current recommendations and clinical judgment [3, 4]. Various studies have focused on the perfect position for radiographs, such as orientation, film/sensor placement and exposure factors. Mistakes in acquiring radiographs may injure diagnostic efficiency and promote misinterpretations, leading to therapeutic failure. In addition, the clinician's attitude regarding radiation safety, compliance with the ALARA (As Low as Reasonably Achievable) principle and knowledge of regulatory paths are key elements in patient protection and (ethical) behavior [5]. Differences between dentists in the use of endodontic radiographic techniques are influenced mainly by academic training, professional experience, availability to advanced imaging technology and the level of continuing education [6].

The absence of this equipment in areas and the lack of updated training for modern imaging compounds contribute to the nonideal use of radiography in endodontic treatment. Moreover, there is a lack of uniform application of protocols, even with guidelines recommended by professional organizations like the American Association of Endodontists (AAE) and the European Society of Endodontology (ESE) being available [7]. With the recent integration of artificial intelligence (AI) and deep learning technologies into endodontics, particularly in radiographic interpretation and diagnostic decision-making, clinicians are now equipped with advanced tools that enhance accuracy and efficiency in treatment planning [8]. Survey-based results are much needed to assess and quantify the existing knowledge, attitudes and practices (KAP) among dental practitioners in this area. By highlighting discrepancies in understanding and practice, such studies may contribute to the design of targeted educational programs, setting standards, and quality improvement work. Furthermore, the findings of KAP surveys benefit dental training centers and associations to review and amend their curriculum and continue education programs [9]. Therefore, it is of interest to evaluate the knowledge, attitude, and practice of endodontic radiographic techniques among dental practitioners.

## Methodology:

This cross-sectional descriptive survey assessed the knowledge, attitude, and practice (KAP) of endodontic radiographic techniques among practicing dentists. The study evaluated dentists' understanding of radiographic principles, attitudes towards using radiographic technologies in endodontics, and routine clinical practices involving radiographic imaging.

## Study design and setting:

A structured, self-administered questionnaire was designed and distributed among licensed dental practitioners in private clinics, government hospitals, and academic institutions. The study lasted three months.

## Study population and sampling:

The target population included general dental practitioners, endodontists, and postgraduate students in dentistry. A convenience sampling method was adopted, with an estimated sample size of 250 participants to ensure adequate representation and statistical reliability. Participants who provided informed consent and met the inclusion criteria were enrolled in the study. Dentists with less than one year of clinical experience and those unwilling to participate were excluded.

## Questionnaire design:

The survey instrument consisted of four sections:

[1] Demographic information - including age, gender, qualification, years of clinical experience, and practice setting.

[2] Knowledge-based questions: These assess familiarity with radiographic techniques, radiation safety protocols, digital imaging systems, and CBCT applications in endodontics.

[3] Attitude-based statements - evaluated using a 5-point Likert scale to gauge perceptions of endodontic radiographs' necessity, safety, and reliability.

[4] Practice-based questions - related to the frequency and types of radiographs used during various endodontic procedures, adherence to the ALARA principle, and use of protective measures such as lead aprons and thyroid collars.

The questionnaire was pre-tested on 20 dentists to ensure clarity, relevance, and reliability. Based on feedback, necessary modifications were made before final distribution.

## Sample size and calculation:

Three hundred eighty-four dental professionals participated in this cross-sectional survey assessing endodontic radiographic techniques' knowledge, attitude, and practice (KAP). The sample size was determined using the standard formula for estimating proportions in descriptive studies: Accordingly, the minimum required sample size was calculated to be 384 participants to ensure representativeness, reduce sampling error, and maintain a 95% confidence level with a 5% margin of error. Participants were selected through a convenience sampling technique from a diverse pool of dental practitioners, including general dentists, endodontists, and postgraduate dental students, to ensure variability in clinical experience and practice settings. This sample size provides sufficient power to detect significant differences and associations within the knowledge, attitude, and practice domains related to endodontic radiographic techniques.

## Data collection:

The questionnaire was distributed in print and electronically via email and online survey platforms (e.g., Google Forms). Participants were informed about the purpose of the study and assured of confidentiality. Completing and submitting the questionnaire was considered implied consent.

## Data analysis:

Collected data were compiled, entered into Microsoft Excel, and analyzed using SPSS software (version 27). Descriptive statistics such as frequencies, percentages, means, and standard deviations were calculated. Inferential statistics, including chi-square tests and ANOVA, assessed associations between demographic variables and KAP scores. A p-value of less than 0.05 was considered statistically significant.

## Results:

A total of 384 dental professionals participated in the study. The study analyzed participants' demographic characteristics, knowledge, attitudes, and practice patterns related to endodontic radiographic techniques. Out of 384 participants, the majority were general dental practitioners (47.9%), followed by postgraduate students (31.5%) and endodontic specialists (20.6%). Most participants had 1-5 years of clinical experience (Table 1). Most participants were aware of RVG radiographs, but only 58.9% were familiar with CBCT applications in endodontics. Awareness of radiation safety protocols was reported by 72.3% of respondents (Table 2). Most (91.1%) believed that radiographs are essential for endodontic diagnosis. However, only 61.5% were confident in interpreting CBCT images. Most participants showed a positive attitude toward adopting advanced imaging when trained (Table 3). Most respondents routinely used RVG in clinical practice. CBCT use was limited to only 39.6% of respondents, usually on referral. Protective measures like lead aprons and thyroid collars were not universally applied (Table 4).

## Discussion:

This study provides a comprehensive overview of the knowledge, attitude and practice (KAP) of endodontic radiographic techniques, particularly RVG and CBCT, among dental professionals in India. The results reveal a clear preference and familiarity with RVG, which was reported as the most commonly used and well-understood modality in endodontic practice. In contrast, although CBCT is acknowledged for its advanced diagnostic capabilities, its use remains relatively limited, mainly due to a lack of training, restricted access, and interpretation challenges. All participants in the current study (100%) were aware of RVG as a radiographic tool, while only 58.9% reported familiarity with CBCT in endodontics. These findings are consistent with previous studies by Krishnamachari [10], which also noted that general dentists were more conversant with conventional radiography than advanced 3D imaging modalities like CBCT. The lower awareness regarding CBCT is likely due to the limited exposure during undergraduate training and a lack of hands-on experience in general practice. The study by Ansari *et al.* (2024) highlights varied levels of knowledge and application of endodontic radiographic techniques among dentists, underscoring gaps in standardized practice. This aligns with your survey findings, emphasizing the need for enhanced training to improve diagnostic accuracy and patient outcomes in endodontics [10]. Despite this, the current study revealed an encouraging attitude toward adopting CBCT technology, with 86.2% of participants willing to receive formal training. This agrees with the findings of Shah *et al.* [11], who emphasized that practitioners may hesitate to use CBCT due to interpretation complexity and radiation concerns. Still, they are receptive to professional development opportunities when supported by guidelines and structured workshops. RVG was routinely used by 96.1% of the respondents, underscoring its role as the cornerstone of radiographic imaging in endodontics. However, only 39.6% of the respondents used CBCT, primarily in complex cases. Deepak *et al.* [12] reported similar findings. RVG was preferred for its ease of use and cost-effectiveness, while CBCT was reserved for advanced diagnostics such as root resorption, complex canal anatomy, or surgical endodontics. Regarding radiation safety, only 69.5% of the respondents consistently used lead aprons and just 57.0% used thyroid collars, which suggests room for improvement. Cheoron *et al.* [13] reported comparable safety practice rates in Saudi Arabia, highlighting the need for stronger adherence to the ALARA principle. Furthermore, with emerging technologies such as artificial intelligence being explored in radiographic interpretation as discussed by Keerthanasree *et al.* [14], there is an urgent need to update training curricula to include CBCT use and its integration with AI-assisted diagnostics.

## Conclusion:

Dental practitioners are familiar with conventional radiographic techniques such as RVG. However, the adoption of advanced technologies like CBCT remains limited due to training gaps and accessibility. Nonetheless, there is a positive attitude toward incorporating these advanced tools, with many practitioners willing to undergo further training. These findings highlight the need for updated education and guidelines to ensure optimal use of radiographic techniques in endodontics.

## Figures and Tables

**Table 1:** Demographic characteristics of participants

**Characteristic**	**Category**	**Frequency (n)**	**Percentage (%)**
Gender	Male	213	55.50%
	Female	171	44.50%
Qualification	BDS	184	47.90%
	MDS - Endodontics	79	20.60%
	Postgraduate Students	121	31.50%
Years of Practice	<1 year	48	12.50%
	1-5 years	191	49.70%
	>5 years	145	37.80%
Type of Practice	Private Clinic	156	40.60%
	Government Hospital	86	22.40%
	Academic Institution	142	37.00%

**Table 2:** Knowledge-based responses

**Knowledge Parameter**	**Response as "YES" (n)**	**Percentage (%)**
Awareness of RVG usage	384	100.00%
Knowledge of digital radiography	327	85.20%
Familiarity with CBCT in endodontics	226	58.90%
Understanding of ALARA/ALADA principles	278	72.30%
Training in radiation protection protocols	249	64.80%

**Table 3:** Attitude-based responses

**Attitude Statement**	**Agree/Strongly Agree (n)**	**Percentage (%)**
Radiographs are essential in endodontic therapy	350	91.10%
I feel confident interpreting digital radiographs	294	76.60%
I feel confident interpreting CBCT images	236	61.50%
I am willing to undergo training in advanced imaging	331	86.20%

**Table 4:** Practice-based responses

**Practice Parameter**	**Yes (n)**	**Percentage (%)**
Use of RVG in routine endodontic cases	369	96.10%
Use of CBCT for complex root canal anatomy	152	39.60%
Use of lead apron during radiographic procedures	267	69.50%
Use of thyroid collar during radiographs	219	57.00%
Following ALARA/ALADA principles in routine practice	289	75.30%
